# A Spectrum Access Based on Quality of Service (QoS) in Cognitive Radio Networks

**DOI:** 10.1371/journal.pone.0155074

**Published:** 2016-05-12

**Authors:** Linbo Zhai, Hua Wang, Chuangen Gao

**Affiliations:** 1 School of Computer Science and Technology, Shandong University, Jinan, China; 2 Shandong Provincial Key Laboratory for Distributed Computer Software Novel Technology, Shandong Normal University, Jinan, China; Nankai University, CHINA

## Abstract

The quality of service (QoS) is important issue for cognitive radio networks. In the cognitive radio system, the licensed users, also called primary users (PUs), are authorized to utilize the wireless spectrum, while unlicensed users, also called secondary users (SUs), are not authorized to use the wireless spectrum. SUs access the wireless spectrum opportunistically when the spectrum is idle. While SUs use an idle channel, the instance that PUs come back makes SUs terminate their communications and leave the current channel. Therefore, quality of service (QoS) is difficult to be ensured for SUs. In this paper, we first propose an analysis model to obtain QoS for cognitive radio networks such as blocking probability, completed traffic and termination probability of SUs. When the primary users use the channels frequently, QoS of SUs is difficult to be ensured, especially the termination probability. Then, we propose a channel reservation scheme to improve QoS of SUs. The scheme makes the terminated SUs move to the reserved channels and keep on communications. Simulation results show that our scheme can improve QoS of SUs especially the termination probability with a little cost of blocking probability in dynamic environment.

## Introduction

Today, internet of things, cloud computing and mobility has brought up too much data for users. It is evident that so much data should be transmitted by the wireless system. On the other hand, the wireless spectrum has been authorized to licensed users while the spectrum utilization is at low rate. To improve the utilization of spectrum resource, cognitive radio technology (CR) [1] has recently emerged as a promising solution. In the cognitive radio system, the licensed users which are called primary users (PUs) are authorized to utilize the wireless spectrum, and the unlicensed users which are called secondary users (SUs) are not authorized to access the wireless spectrum. Cognitive radio allows SUs access licensed spectrum opportunistically only when the spectrum is not used by PUs. While SUs use an idle channel, the instance that PUs come back makes SUs terminate their communications and leave the current channel. When this occurs, SUs’ transmission is terminated compulsorily. The quality of service (QoS) is difficult to be ensured for SUs.

In a wireless system, PUs may have much data to transmit. Therefore, PUs utilize licensed spectrum frequently. It leads to high probability of SUs’ forced termination. The quality of service (QoS) of SUs is difficult to be ensured. In the cognitive radio system, there are numerous related literatures for QoS of SUs. In distributed cognitive radio networks, QoS for delay-sensitive applications is researched in [2]. Considering QoS requirements of SUs, cross-layer methods are proposed to allocate the resource reasonably in [3, 4]. In [5, 6], the analytical model is proposed to evaluate the SUs’ performance in cognitive radio networks. Considering energy effect, the authors of [7] propose an energy-efficient handoff strategy using the partially observable Markov decision process. In [8], spectrum access strategy with an α-Retry policy is proposed a to enhance QoS for SUs. To satisfy the delay requirement in the cognitive radio system, the authors propose a novel component carrier configuration and switching scheme for real-time traffic in [9]. From the spectrum resource management perspective, a comprehensive analytical framework based on queueing theory is proposed to analyze QoS of SUs in [10]. And there are some related work in [11–13].

The aforementioned researches do not consider QoS in a cognitive radio system where SUs’ transmission is often terminated by PUs. In this paper, QoS of SUs is investigated in the cognitive radio system. In the system, SUs access the wireless spectrum opportunistically when the spectrum is not used by PUs. However, while SUs use an idle channel, the instance that PUs come back to the channel makes SUs terminate their communications and leave the current channel. We first propose an analysis model to derive QoS of SUs such as blocking probability, completed traffic and termination probability of SUs. To improve QoS of SUs especially the forced termination probability, then we propose a channel reservation scheme for SUs. This scheme makes the terminated SUs move to the reserved channels and keep on transmission. Simulation results show that our proposed scheme can improve QoS of SUs especially the termination probability with a little cost of blocking probability in dynamic environment.

The rest of the paper is organized as follows: In section 2, the analysis model is presented to obtain QoS of SUs. In section 3, a channel reservation scheme is proposed to improve QoS of SUs. In Section 4, simulation results show that our scheme reduces termination probability with the cost of a litter increase of blocking probability. Finally, this paper is concluded in Section 5.

## The Analysis Model

**Fig 1:**
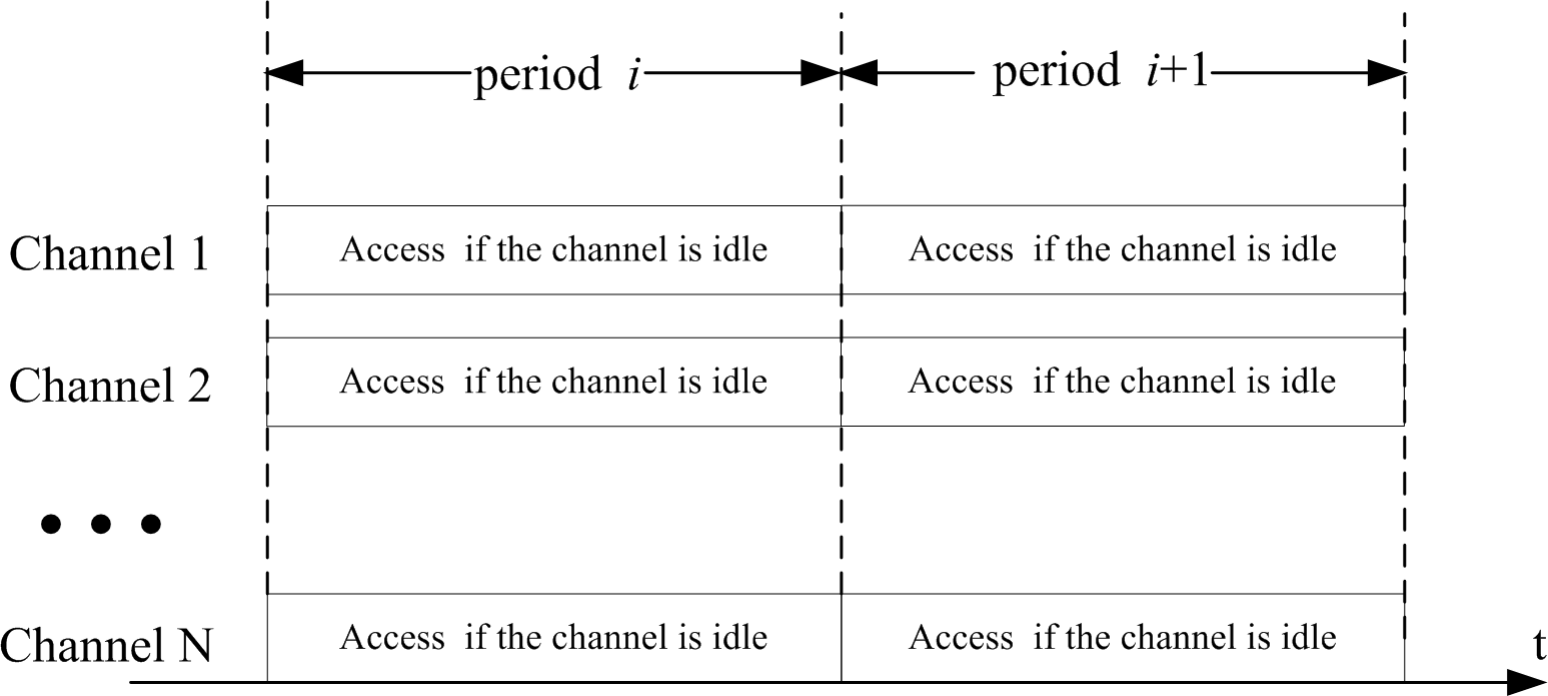
SUs’ periodic sensing and access.

In the system, there are *N* channels with equal bandwidth. When PUs do not utilize one or several channels, SUs could access the idle channels for transmission. Therefore, it is necessary to sense the channels periodically for SUs which have data to transmit as in Fig 1. At the beginning of each period, SUs perform spectrum sensing to find out whether the channel is used by the PUs or not. We assume that spectrum sensing is perfect and sensing period is negligible. For SUs, when they notice there are some idle channels after spectrum sensing, a SU can only access one idle channel until the next sensing. When a PU comes back to this channel with a SU’s data, the SU must leave this channel and the SU’s transmission is terminated. In this section, QoS of the SUs is analyzed in terms of blocking probability, the completed traffic and termination probability of SUs.

It is assumed that the SUs’ traffic arrival and departure streams follow Poisson random processes. In the whole system, let *λ* denote the arrival rate of SUs, which means how many SUs want to transmit data in unit time, and *μ* denote the departure rate of SUs, which means the average time of each SU transmission is 1/*μ*. The whole input traffic of SUs is *A* = *λ*/*μ*. When there are *n* channels are idle (unused by PUs), considering a SU can only access one idle channel, there are *n* SUs which can use the idle channels at most. If more than *n* SUs want to access channels in the cognitive radio system, some SUs’ traffic may be blocked. According to the Erlang loss formula, the probability of SU traffic blocking is obtained as

$$B=\frac{A^{n}/n!}{\sum_{i=1}^{n}A^{i}/i!}$$ (1)

Considering the traffic blocking, the completed traffic of SUs is *A*(1−*B*) if no PUs use channels during SUs’ transmission. However, PUs may return to these channels where SUs are transmitting data. SUs must leave these channels and their transmissions are terminated. Therefore, *A*(1−*B*) does not denote the real completed traffic.

In each channel, we assume the PUs’ traffic arrival and departure streams follow Poisson random processes. Let *λ*_1_ denote the arrival rate of PUs, *μ*_1_ denote the departure rate of PUs for each channel, and *T* denote the duration of one period for SUs. For each idle channel which is used by SUs, the probability that a PU comes during SUs’ transmission is $p=1-e^{-\lambda_{1}T}$. When PUs’ traffic occurs in *j* channels where SUs are transmitting data, SUs’ traffic is terminated in the *j* channels and only *n* − *j* channels can be used by SUs. The probability that PUs’ traffic occurs in *j* channels is

$$P\{\text{PUs return in}\;j\;\text{channels}\}=\left(\begin{array}{c} j\\ n \end{array}\right)\;p^{j}{\left(1-p\right)}^{n-j}$$ (2)

During a period, when PUs’ traffic comes in one out of *n* channels, SUs can only use *n*-1 channels after PUs’ traffic comes. According to the Erlang loss formula, the probability of SU traffic blocking can be obtained as $B_{1}=A^{n-1}/(n-1)!/\sum_{i=1}^{n-1}A^{i}/i!$. Before PUs’ traffic comes, SU traffic blocking probability is still *B*. The probability density function of the duration between the beginning of the period and the moment PUs’ traffic comes is $f(t)=e^{-\lambda_{1}t}$. In this case, the corresponding completed traffic of SUs is

$$Tra(1)=\int_{0}^{T}e^{-\lambda_{1}t}[A\frac{t}{T}(1-B)+A\frac{T-t}{T}(1-B_{1})]dt$$ (3)

During a period, when PUs’ traffic comes in two out of *n* channels, it is assumed that PUs’ traffic comes first in one channel and second in another channel. Let *t*_1_ denote the duration between the beginning of the period and the moment PUs’ traffic comes first, and *t*_2_ denote the duration between the beginning of the period and the moment PUs’ traffic comes second (*t*_1_ ≤ *t*_2_). For each channel, the PUs’ traffic is independent. Therefore, the probability density function of (*t*_1_, *t*_2_) is $f(t_{1},t_{2})=f(t_{1})f(t_{2})=e^{-\lambda_{1}t_{1}}e^{-\lambda_{1}t_{2}}$. When no PUs’ traffic comes, SU traffic blocking probability is *B*. When PUs’ traffic comes first in one out of *n* channels, SU traffic blocking probability is *B*_1_. When PUs’ traffic comes second in another one out of *n* channels, according to the Erlang loss formula, SU traffic blocking probability is *B*_2_ which can be obtained as $B_{2}=A^{n-2}/(n-2)!/\sum_{i=1}^{n-2}A^{i}/i!$. In this case, the corresponding completed traffic of SUs is

$$Tra(2)=\int_{0}^{T}\int_{t_{1}}^{T}f(t_{1},t_{2})[A\frac{t_{1}}{T}(1-B)+A\frac{t_{2}-t_{1}}{T}(1-B_{1})+A\frac{T-t_{2}}{T}(1-B_{2})]dt_{2}dt_{1}$$ (4)

During a period, when PUs’ traffic comes in three or more than three channels, the corresponding completed traffic *Tra*(*j*) (*j* ≥ 3) of SUs can be calculated by the similar method.

Therefore, the real completed traffic of SUs is

$$Tr=P\{\text{PUs return in}\;0\;\text{channels}\}A(1-B)+\sum_{j=1}^{n}P\{\text{PUs return in}\;j\;\text{channels}\}Tra(j)$$ (5)

Compared to the completed traffic of SUs equaling *A*(1−*B*) without PUs’ traffic coming, the terminated SUs’ traffic is *A*(1−*B*)−*Tr*. Therefore, termination probability of SUs can be obtained as

$$P_{ter}=\frac{A(1-B)-Tr}{A(1-B)}$$ (6)

## Channel Reservation

In a wireless system, PUs have too much data and they come to channels irregularly. SUs’ transmission is terminated frequently. To ensure the QoS of SUs, the channel reservation scheme is proposed. When there are reserved idle channels, terminated SUs’ communications can move immediately to reserved channels to avoid communications termination. Channel reservation is proposed in this section. With the channel reservation scheme, we analyze QoS of the SUs in terms of blocking probability, the real completed traffic and termination probability of SUs.

Let *m* denote maximum channels that SUs can utilize, which means no more than *m* channels are assigned to SUs no matter how many idle channels there are. The remaining idle channels are reserved. Terminated SUs can move to the reserved channels. Obviously, if the current number of idle channels is less than *m*, SUs can only access the current idle channels.

We assume that the SUs’ traffic arrival and departure streams follow Poisson random processes. In the whole system, let *λ* denote the arrival rate of SUs, and *μ* denote the departure rate of SUs. The whole input traffic of SUs is *A* = *λ*/*μ*. When there are *n* channels are idle (unused by PUs), SUs cannot access all these idle channels but only *m* channels (*m* < *n*) instead since the channel reservation scheme is used. According to the Erlang loss formula, the probability of SU traffic blocking under the reservation condition is obtained as

$$Br=\frac{A^{m}/m!}{\sum_{i=1}^{m}A^{i}/i!}$$ (7)

The completed traffic of SUs is *A*(1−*Br*) without PUs’ traffic coming, and there are *n*−*m* idle channels which are reserved. However, PUs may return to these channels where SUs are transmitting data. When PUs want to utilize one or several channels of *m* channels which SUs are using, SUs must leave these channels and move immediately to reserved channels. When there are remaining idle channels, forced termination will not occur. Therefore, during a period, when PUs’ traffic comes in less than *n*−*m*+1 out of *n* channels, no forced termination occurs. After all reserved channels are used, forced termination may happen.

During a period, when PUs’ traffic comes in *n*−*m*+1 out of *n* channels, all reserved channels are used and SUs’ traffic in one channel must be terminated. SUs can only use *m*-1 channels after PUs’ traffic comes. According to the Erlang loss formula, the probability of SU traffic blocking can be calculated as $Br_{1}=A^{m-1}/(m-1)!/\sum_{i=1}^{m-1}A^{i}/i!$. Before the *n*−*m*+1th PUs’ traffic comes, SU traffic blocking probability is still *Br*. In each channel, it is assumed the PUs’ traffic arrival and departure streams follow Poisson processes. Let *λ*_1_ denote the arrival rate of PUs, *μ*_1_ denote the departure rate of PUs for each channel, and *T* denote the duration of one period for SUs. The probability density function of the duration between the beginning of the period and the moment the *n*−*m*+1th PUs’ traffic comes is $f(t)=e^{-\lambda_{1}t}$. In this case, the corresponding completed traffic of SUs is

$$Trar(n-m+1)=\int_{0}^{T}e^{-\lambda_{1}t}[A\frac{t}{T}(1-Br)+A\frac{T-t}{T}(1-Br_{1})]dt$$ (8)

During a period, when PUs’ traffic comes in *n*−*m*+2 out of *n* channels, SUs’ traffic in two channels must be terminated. For the two channels, it is assumed that PUs’ traffic comes first in one channel and second in another channel. Let *t*_1_ denote the duration between the beginning of the period and the moment PUs’ traffic comes in one channel first, and *t*_2_ denote the duration between the beginning of the period and the moment PUs’ traffic comes in another channel second (*t*_1_ ≤ *t*_2_). For each channel, the PUs’ traffic is independent. Therefore, the probability density function of (*t*_1_, *t*_2_) can be obtained as $f(t_{1},t_{2})=f(t_{1})f(t_{2})=e^{-\lambda_{1}t_{1}}e^{-\lambda_{1}t_{2}}$. When PUs’ traffic comes in *n*−*m* channels, SU traffic blocking probability is *Br*. When PUs’ traffic comes in *n*−*m*+1 channels, SU traffic blocking probability is *Br*_1_. When PUs’ traffic comes in *n*−*m*+2 channels, SU traffic blocking probability is *Br*_2_ which can be obtained as $Br_{2}=A^{m-2}/(m-2)!/\sum_{i=1}^{m-2}A^{i}/i!$ according to the Erlang loss formula. In this case, the corresponding completed traffic of SUs is

$$Trar(n-m+2)=\int_{0}^{T}\int_{t_{1}}^{T}f(t_{1},t_{2})[A\frac{t_{1}}{T}(1-Br)+A\frac{t_{2}-t_{1}}{T}(1-Br_{1})+A\frac{T-t_{2}}{T}(1-Br_{2})]dt_{2}dt_{1}$$ (9)

During a period, when PUs’ traffic comes in more than *n*−*m*+2 channels, the corresponding completed traffic *Trar*(*j*) (*j*≥*n*−*m*+3) of SUs can be calculated by the similar method.

Therefore, the whole completed traffic can be obtained as

$$Trr=\sum_{j=0}^{n-m}P\{\text{PUs return in}\;j\;\text{channels}\}A(1-Br)+\sum_{j=n-m+1}^{n}P\{\text{PUs return in}\;j\;\text{channels}\}Trar(j)$$ (10)

Forced terminating probability can be derived as

$$P_{terr}=\frac{A(1-Br)-Trr}{A(1-Br)}$$ (11)

## Numerical Results and Discussions

In this section, QoS of SUs with channel reservation is analyzed in terms of blocking probability, the real completed traffic and termination probability of SUs according to the analytical model above. And the analytical results are validated by simulations.

**Fig 2:**
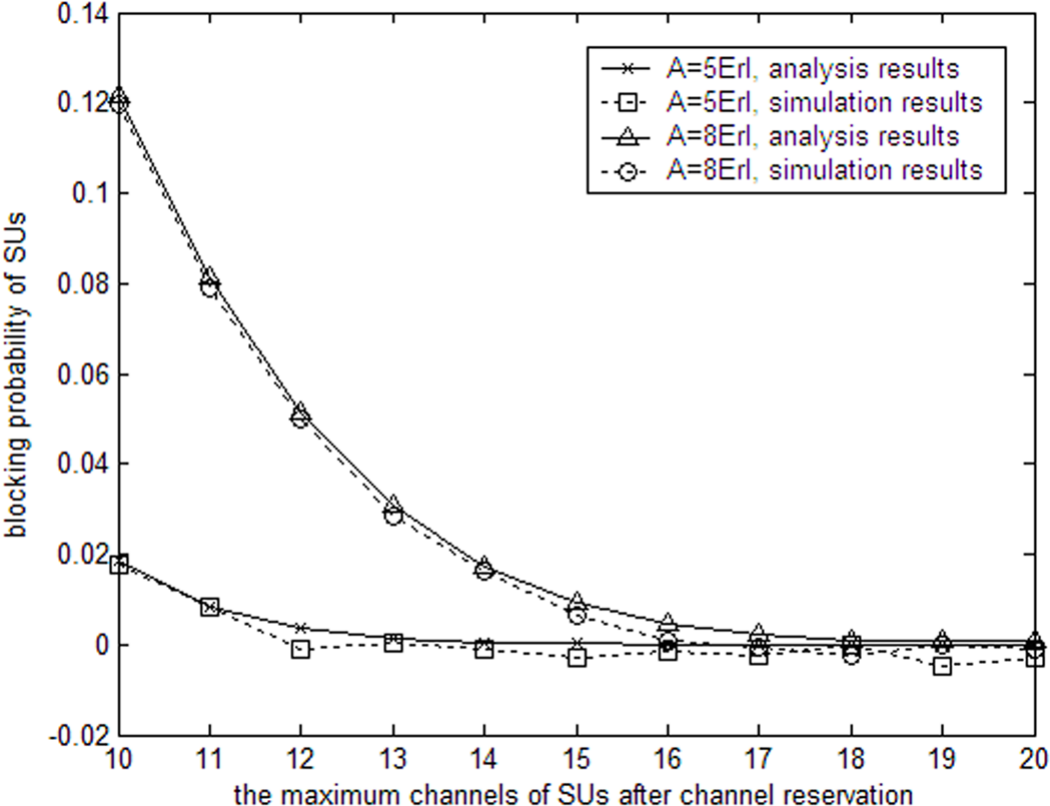
SU traffic blocking probability with allowable channels.

**Fig 3:**
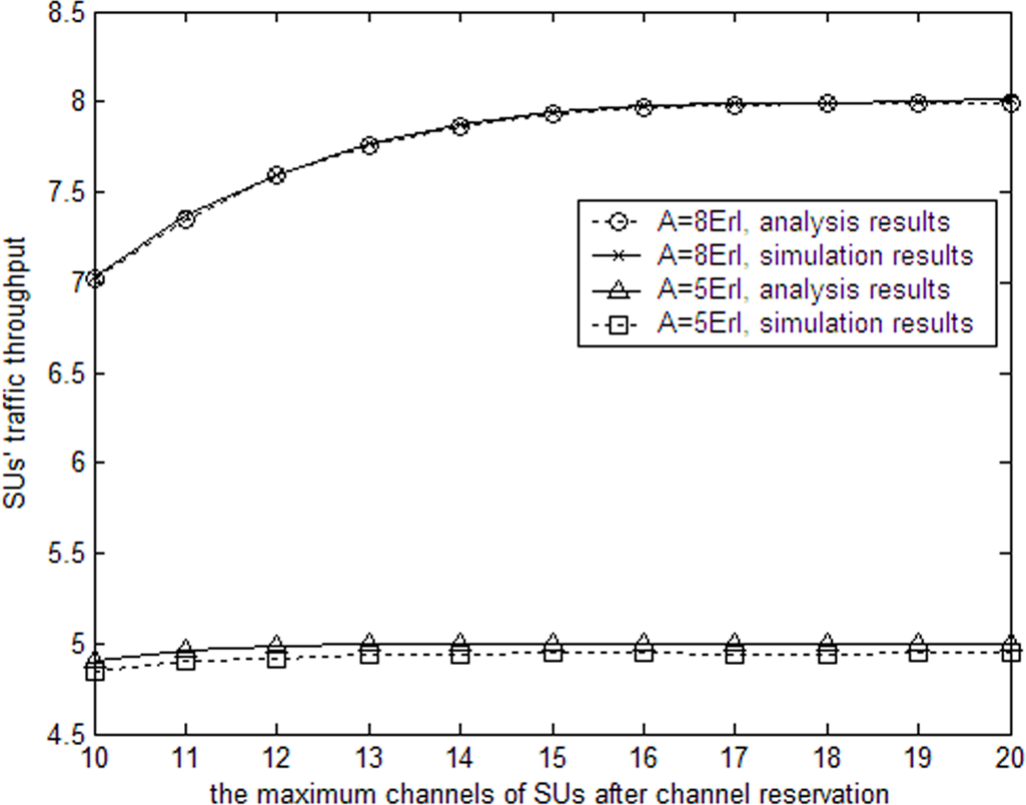
SUs’ completed traffic throughput with allowable channels.

The parameters of Figs 2 and 3 are as follows. For SUs, the duration of one period is *T* = 20ms. There are *N* = 30 channels in the system. The departure rate of PUs for each channel is *μ*_1_ = 0.02s^−1^. And the whole input traffic of PUs is 30Erl. Erl is unit of input traffic and completed traffic. It can be obtained by arrival rate dividing departure rate. In Fig 2, the probability of SU traffic blocking is analyzed when the input traffic of SUs equals 5Erl and 8Erl, respectively. When there are idle channels, SUs can access these channels. Our channel reservation scheme limit the maximum allowable channels (denoted by *m* in section III) that SUs can utilize. Except the maximum allowable channels that SUs access, the remaining idle channels are reserved for terminated SUs to move to continue their communications. Therefore, as maximum allowable channels increases, the number of reserved channels decreases. Since more idle channels are used by SUs, the SU traffic blocking probability decreases.

Fig 3 depicts the completed traffic of SUs with the number of maximum allowable channels. When the input traffic of SUs equals 5Erl, a few idle channels can finish the transmission of SUs’ traffic because the input traffic of SUs is very low. Therefore, as maximum allowable channels for SUs increases, the completed traffic throughput of SUs increases slightly. When the input traffic of SUs equals 8Erl, the input traffic increases and a few idle channels cannot satisfy SUs’ traffic. As maximum allowable channels increases, the completed traffic throughput of SUs increases noticeably than that of SUs with low input traffic.

**Fig 4:**
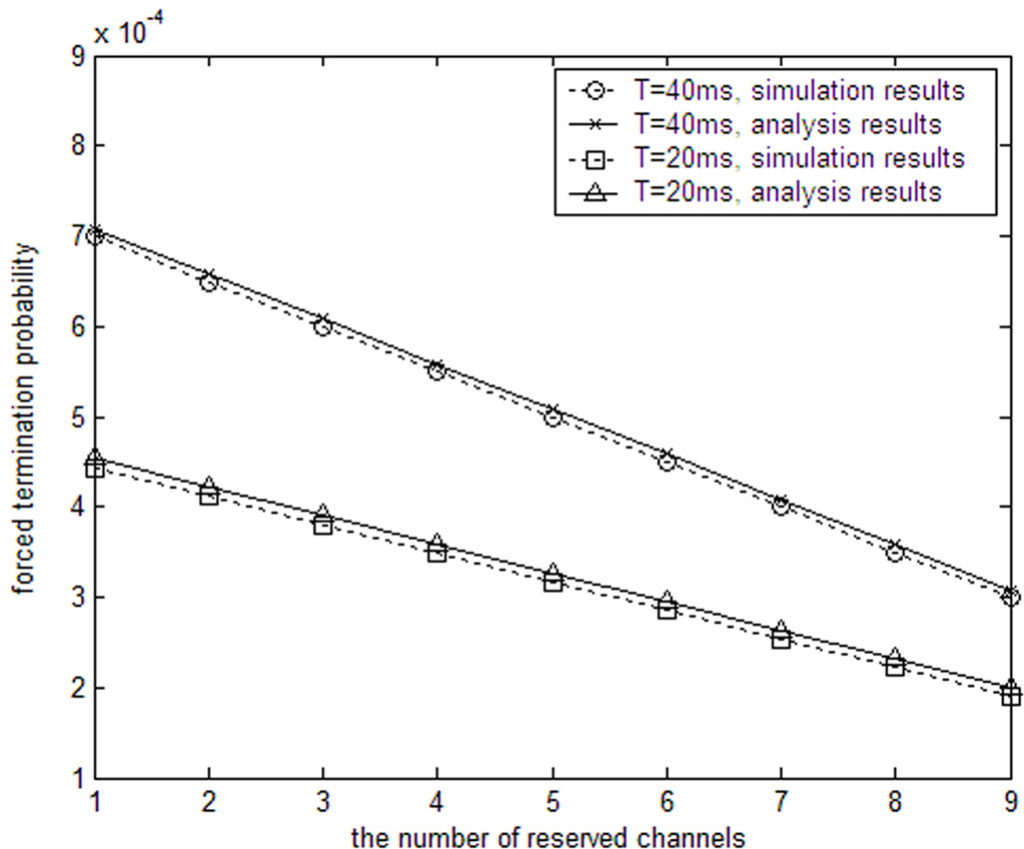
SUs’ forced termination probability with reserved channels.

In Fig 4, the number of channels is *N* = 30, the departure rate of PUs is *μ*_1_ = 0.02s^−1^, and the whole input traffic of PUs is 30Erl. When the duration of one period for SUs equals 20ms and 40ms, the SU traffic termination probability decreases as reserved channels increases. The reason is that more channels are reserved for SUs to avoid termination. Therefore, channel reservation can decrease the forced termination probability while increase the blocking probability. In general, channel reservation is advantageous since users are more sensitive to be terminated of an ongoing transmission than to be rejected of a new transmission.

## Conclusion

In this paper, an analysis model is presented to derive the quality of service (QoS) for SUs such as blocking probability, completed traffic and termination probability of SUs in the wireless system. Since PUs have too much data to transmit and often access the channels, the quality of service of SUs is difficult to ensured, especially the forced termination probability. Therefore, a channel reservation scheme for SUs is proposed to improve the quality of service of SUs. This scheme makes the terminated SUs move to the reserved channels and keep on transmission. Simulation results show that our scheme can improve QoS of SUs especially the termination probability with a little cost of blocking probability in dynamic environment.
